# Pain Mirrors: Neural Correlates of Observing Self or Others’ Facial Expressions of Pain

**DOI:** 10.3389/fpsyg.2018.01825

**Published:** 2018-10-02

**Authors:** Francesca Benuzzi, Fausta Lui, Martina Ardizzi, Marianna Ambrosecchia, Daniela Ballotta, Sara Righi, Giuseppe Pagnoni, Vittorio Gallese, Carlo Adolfo Porro

**Affiliations:** ^1^Department of Biomedical, Metabolic and Neural Sciences, University of Modena and Reggio Emilia, Modena, Italy; ^2^Center for Neuroscience and Neurotechnology, University of Modena and Reggio Emilia, Modena, Italy; ^3^Unit of Neuroscience, Department of Medicine and Surgery, University of Parma, Parma, Italy

**Keywords:** pain, empathy, mirror, facial expression, self

## Abstract

Facial expressions of pain are able to elicit empathy and adaptive behavioral responses in the observer. An influential theory posits that empathy relies on an affective mirror mechanism, according to which emotion recognition relies upon the internal simulation of motor and interoceptive states triggered by emotional stimuli. We tested this hypothesis comparing representations of self or others’ expressions of pain in nineteen young healthy female volunteers by means of functional magnetic resonance imaging (fMRI). We hypothesized that one’s own facial expressions are more likely to elicit the internal simulation of emotions, being more strictly related to self. Video-clips of the facial expressions of each volunteer receiving either painful or non-painful mechanical stimulations to their right hand dorsum were recorded and used as stimuli in a 2 × 2 (Self/Other; Pain/No-Pain) within-subject design. During each trial, a 2 s video clip was presented, displaying either the subject’s own neutral or painful facial expressions (Self No-Pain, SNP; Self Pain, SP), or the expressions of other unfamiliar volunteers (Others’ No-Pain, ONP; Others’ Pain, OP), displaying a comparable emotional intensity. Participants were asked to indicate whether each video displayed a pain expression. fMRI signals were higher while viewing Pain than No-Pain stimuli in a large bilateral array of cortical areas including middle and superior temporal, supramarginal, superior mesial and inferior frontal (IFG) gyri, anterior insula (AI), anterior cingulate (ACC), and anterior mid-cingulate (aMCC) cortex, as well as right fusiform gyrus. Bilateral activations were also detected in thalamus and basal ganglia. The Self vs. Other contrast showed signal changes in ACC and aMCC, IFG, AI, and parietal cortex. A significant interaction between Self and Pain [(SP vs. SNP) >(OP vs. ONP)] was found in a pre-defined region of aMCC known to be also active during noxious stimulation. These findings demonstrate that the observation of one’s own and others’ facial expressions share a largely common neural network, but self-related stimuli induce generally higher activations. In line with our hypothesis, selectively greater activity for self pain-related stimuli was found in aMCC, a medial-wall region critical for pain perception and recognition.

## Introduction

Observing someone expressing emotion or someone in pain tends to elicit empathy, broadly defined as “the experiencing of an affective or sensory state similar to that shown by a perceived individual, where one is aware as to whether the source of the state is oneself or another” (Fan et al., 2011).

There is still debate as to whether empathy represents a “self-centered” or an “other-oriented” response. On the one hand, the observation of another’s distress can evoke the aversive experience of personal distress, a self-oriented response (Davis, 1983; Eisenberg, 2000) associated with fear, uncertainty, emotional vulnerability, anxiety, and negative affect (Batson et al., 1987). On the other hand, seeing someone in pain can also trigger empathic concern for his/her wellbeing, an other-oriented response (Batson et al., 1987; Eisenberg, 2000). Along the same lines, empathy can convey both the sense of “feeling as” another person (to feel as another feels), and “feeling for” him/her, a concept similar to pity, sympathy, or compassion (Batson, 2011). These mental processes are not necessarily mutually exclusive, and could likely occur at the same time.

Faces are a special class of stimuli in that they are one of the most expressive perceptual signals for the emotional state of another person. Indeed, facial expressions play a central role in social interactions and can elicit rapid responses in the observer (Craig et al., 2001).

Although several researches have evaluated the behavioral correlates of facial expressions of pain (Craig et al., 2001), the neural substrates for the processing of painful facial expressions have not been investigated in depth, and few functional neuroimaging studies on empathy for pain have used expressive faces as stimuli (Botvinick et al., 2005; Lamm et al., 2007; Saarela et al., 2007; Budell et al., 2010). Previous studies on the neural basis of empathy for pain have predominantly used visual tasks where participants watched another person’s body parts in painful situations (see Fan et al., 2011 for a review). Functional magnetic resonance (fMRI) studies consistently showed overlapping neural activations for the direct experience of pain and for the observation of the pain of others (Lamm et al., 2011). Specifically, two areas involved in nociceptive processing – the anterior insula (AI) and the anterior mid-cingulate cortex (aMCC) – usually respond not only when we experience pain, but also when we observe painful stimuli in others (Singer et al., 2004, 2006; Saarela et al., 2007; Ochsner et al., 2008; Lamm et al., 2011; Zaki et al., 2016). Interestingly, these same areas (aMCC and AI) have been found to be active in the few functional neuroimaging studies on empathy that used facial expressions of pain as stimuli (Botvinick et al., 2005; Lamm et al., 2007; Saarela et al., 2007; Budell et al., 2010).

These findings support an *embodied simulation perspective*, according to which emotion recognition relies upon the internal simulation of motor and interoceptive states triggered by emotional stimuli (Gallese, 2003; Gallese et al., 2004; Henrich et al., 2007): embodied simulation processes are thought to be supported by mirror mechanisms by which the perception of another person’s action, sensation or emotion triggers the activation of the perceiver’s own motor, viscero-motor or somatosensory representation of that specific action, emotion, or sensation.

In this line of thought, the presence of a shared neural network activated both for experiencing and for observing the same emotion has been demonstrated for disgust, i.e., the same portion of the AI, subserving the subjective experience of disgust, is also activated when observing facial expressions of disgust in others (Wicker et al., 2003). A similar hypothesis of shared neural representations has been advanced regarding the neural correlates of pain-related events – either actually experienced pain or the observation of pain in others – in the mid-insula and MCC (Corradi-Dell’Acqua et al., 2011, 2016). For instance, the results of a multivariate pattern analysis fMRI study provide strong (although still indirect) evidence in favor of the recruitment of the same neuronal populations for the two pain-related conditions; this supports the idea that the same distributed cortical network – a shared neural representation – codes painful events, regardless of the subject who is physically affected (Corradi-Dell’Acqua et al., 2011). Few data have been collected with respect to the spatial overlap of cortical representations of self-experienced and observed emotions. In the present study, we aimed at evaluating if pain recognition in others relies upon activity in areas known to be engaged when experiencing pain (Lui et al., 2008; Duerden and Albanese, 2013; Wager et al., 2013; Favilla et al., 2014). We also hypothesized that if empathy relies on an affective mirror mechanism, based on implicit reactivation of motor and interoceptive aspects of pain, self-expressions of pain, despite their lower ecological plausibility, would represent more potent stimuli, given their greater congruency to self-related representations. Therefore, we compared the neural activations related to implicit processing of self and others’ faces, during the presentation of expressions of pain in young female volunteers. To this end, we used video-clips presenting the subject’s own neutral or painful facial expressions, or the neutral/painful expressions of unfamiliar others displaying a comparable emotional intensity, while asking volunteers to judge whether each viewed facial expression was painful or not.

In particular, we evaluated the interaction effect of Self and Pain on BOLD activity in the two brain regions involved in empathy for pain and pain perception, the AI and the aMCC.

## Materials and Methods

### Participants

Twenty-seven right-handed healthy women (mean age = 21.3, *SD* = 1.6; mean school age = 13. 5, *SD* = 1.5) with no history of neurological or psychiatric diseases took part in the study. We decided to use only female volunteers in order to have a homogeneous experimental population, given the well-known differences between genders both in expressivity (LaFrance and Banaji, 1992; Hall et al., 2000), and in empathic behavior (Klein and Hodges, 2001; Singer et al., 2006). Handedness was assessed by means of the Edinburgh Inventory (Oldfield, 1971). All participants gave their written informed consent to take part in the study, which had been previously approved by the local Ethics Committee. The experiment was also conducted in accordance with the ethical standards of the 2013 Declaration of Helsinki.

### Personality Assessment

Participants completed the Italian version of the following questionnaires:

• Empathy questionnaires (Empathy Quotient-EQ, Preti et al., 2011; Interpersonal Reactivity Index-IRI, Albiero et al., 2006; Toronto Alexithymia Scale 20 – TAS 20, Caretti et al., 2005).
• Personality questionnaires (Behavioral Inhibition Scale/Behavioral Activation Scale – BIS/BAS, Carver and White, 1994).
• State Trait Anxiety Inventory – STAI I–II (Sanavio et al., 1997).
• Pain Catastrophizing Scale – PCS (Monticone et al., 2012).

Moreover, volunteers were tested for interoceptive accuracy as measured through the heartbeat perception task (Schandry, 1981). Interoception, the individual sensitivity to physiological stimuli originating inside of the body, is one of the most relevant aspects of self-experience and may influence the perception and evaluation of emotional stimuli (Pollatos et al., 2007; Dunn et al., 2010). In the present study, interoceptive accuracy was assessed as described in Ardizzi et al. (2016) and Ambrosecchia et al. (2017). Briefly, participants had to silently count their own heartbeats in four different time intervals (25, 35, 45, and 100 s) presented in random order and triggered by audio-visual start and stop cues. A brief training period (15 s) preceded the four intervals. During the task, no feedback on the length of the counting phases or the quality of their performance was given and participants were not permitted to take their pulses. Heartbeats were recorded using three Ag/AgCl pre-gelled electrodes (ADInstruments, United Kingdom) with a contact area of 10 mm diameter placed on the wrists of the participants in an Einthoven’s triangle configuration monitoring ECG (Powerlab and OctalBioAmp 8/30, ADInstruments, United Kingdom). The ECG was sampled at 1 KHz and filtered online by the mains filter, which has a negligible distorting effect on ECG waveforms. The R-wave peak of the ECG was detected for each sequential heartbeat. The heartbeat perception score was calculated as the mean score of the four separated heartbeat perception intervals according to the following transformation (Schandry, 1981; Pollatos et al., 2008):

$$\frac{1}{4}\sum \left(1-\frac{\left(|\mathrm{recorded beats}-\mathrm{counted beats}|\right)}{\mathrm{recorded beats}}\right)$$

According to this transformation, heartbeat perception score varies between 0 and 1, with higher scores indicating smaller differences between objectively recorded and subjectively counted heartbeats (i.e., higher interoceptive accuracy).

### Stimuli Recording and Validation

We recorded video clips of each volunteer’s facial expressions while they were receiving either painful or non-painful mechanical stimulations to their right hand. Participants were explicitly told that they would be video-recorded and that they would perceive a painful or a tactile stimulation. During the stimuli-recording phase, they sat comfortably in front of a uniform gray background, wearing a white coat covering their personal clothing. They were also asked to remove make-up, personal jewelry, glasses, or other distinctive elements.

A video camera (Sony HDD handycam DCR-SR32, spatial resolution 720 × 576 pixels) was mounted on a tripod about 1.5 m in front of the participants, at eye level. Environmental settings (e.g., room light, camera distance, sitting position) were kept constant across volunteers. Participants were instructed to ignore the camera and react as they naturally would. Painful (P) and non-painful (NP) stimuli were manually delivered by an experimenter to the right hand dorsum by means of a mechanical stimulator with disposable tips, custom-built in our laboratory. The mechanical stimulator included an aluminum hollow cylinder containing a sliding brass weight connected to a plastic tip, on which either a disposable stainless-steel wire (0.2 mm section) or a foam-rubber tip (approximate diameter: 2 mm) could be mounted for P and NP stimulation, respectively. After each stimulation participants were instructed to rate the evoked sensory intensity using a numerical 0–10 scale, where 0 denoted “no pain” and 10 denoted “the maximum imaginable intensity of pain.” For each volunteer, the brass weight was individually selected, by means of a few test trials, so that the tip would induce a moderate pain sensation (3–4 on a 0–10 scale) for P stimuli and a pure tactile sensation (0 on a 0–10 scale) for NP stimuli.

For each participant, we extracted forty video clips lasting 2 s, one for each stimulation (20 depicting painful and 20 depicting neutral expressions) from their recorded videos using the software VirtualDubMod^1^.

We performed a first rough screening of the resulting 1,080 videos, to eliminate those that were obviously ambiguous. The remaining 329 video clips were presented to a group of three female evaluators from the University staff/faculty in order to select those that were clearly painful or clearly neutral. We chose not naive evaluators because they are better qualified to point out possible problems regarding the stimuli. The evaluators were asked to independently rate each video clip using two separate numerical 0–10 scales, one for the intensity of the facial expression and one for the putative intensity of the pain experienced by participants. Based on these ratings, a minimum of four and a maximum of six videos (those that had received the highest pain intensity scores) were selected for each participant for the fMRI study. Video clips with a mean score <1 were excluded from the P category. The mean intensity score of the selected P videos was 2.81 ± 1.24; range 1–6.

This *a-priori* stimulus categorization served only to increase the probability to find stimuli which could be highly recognizable as painful, but did not affect the following analysis of the fMRI data, which was carried out considering the subjective evaluation given by each single participant (see below, 2.6 fMRI data acquisition and analysis).

Only participants having at least four P videos underwent the fMRI study. For each participant, the same number of N stimuli was randomly chosen among the videos rated 0. Overall, a final set was selected, comprising 228 (114 P and 114 N) video clips from 22 out of 27 volunteers. Because our experimental design requires the observation of the subject’s own facial expressions of pain (see below), the 5 volunteers for whom no P video clips was selected were not admitted to the fMRI study.

### fMRI Experimental Design

An event-related paradigm was employed; it comprised four runs, each composed of eighteen trials, for a total of seventy-two trials. Each trial involved the presentation of a 2 s video clip depicting either:

• the participant’s own neutral facial expressions (self no-pain, SNP);
• neutral facial expressions from one out of three unfamiliar individuals (others’ no-pain, ONP);
• the participant’s own painful facial expressions (self-pain, SP);
• painful facial expressions from one out of three unfamiliar individuals (others’ pain, OP).

For each participant, OP videos were selected in order to match the mean intensity of her painful facial expressions, according to the mean rating provided by the three evaluators.

**FIGURE 1:**
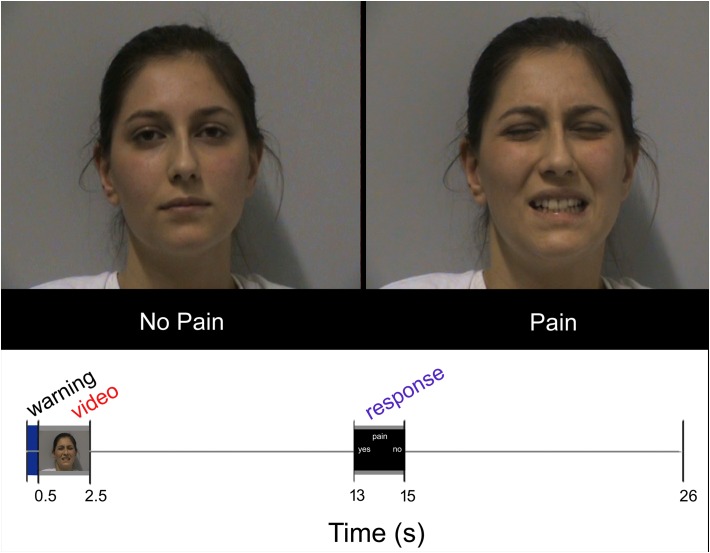
Top: Sample frames extracted from two video-clips representing NP and P stimuli, respectively. Bottom: Experimental design. Temporal sequence of events during each trial. Written informed consent was obtained from the pictured participant.

Each participant underwent 18 trials for each condition (SNP, ONP, SP, and OP); all conditions were arranged in pseudorandom order within each run. In each trial, the volunteers were asked to watch the video clip carefully and, after a 10.5 s interval, to indicate whether the expression was painful or not. Examples of frames taken from the video clips are presented in **Figure 1**, top. Responses were given by pressing one of two buttons of a response box (Current Designs Inc.^2^).

Each trial lasted 26 s and began with a brief (500 ms) change of the background color (from black to blue) as a visual warning cue. Then the following events ensued: stimulus presentation (2 s), black screen (10.5 s), response (2 s), black screen (11 s; **Figure 1**, bottom). The warning cue was introduced to prompt participants about the upcoming appearance of the video clip, but did not provide any hint about the stimulus type. Stimuli were presented via the Esys-fMRI (*Invivo* Corporation, Gainesville, FL, United States) remote display, timed by a custom-made software developed in Visual Basic 6^3^. Accuracy and response times were collected during the scanning sessions by means of the same custom-made software.

### Behavioral Data Analyses

Accuracy and response times were computed for each volunteer separately for the four conditions of interest (SP, SNP, OP, and ONP) and compared using the Friedman test. A value of *p* < 0.05 was taken as the significance difference threshold.

### fMRI Data Acquisition and Analysis

Functional MRI data were acquired with a Philips Achieva MRI system at 3 T and a BOLD-sensitive gradient-echo echo-planar sequence [repetition time (TR): 2,000 ms; echo time (TE): 30 ms; field of view: 240 mm; 80 × 80 matrix; 35 transverse slices, 3 mm each with a 1 mm gap]. A high-resolution T1-weighted anatomical image was also acquired for each subject to allow anatomical localization and spatial standardization (TR: 9.9 ms; TE: 4.6 ms; 170 sagittal slices; voxel size: 1 mm × 1 mm × 1 mm). The Matlab 7.11 and SPM12 (Wellcome Department of Imaging Neuroscience, London, United Kingdom) software were used for data analysis.

Three out of the 22 participants performing the fMRI experiment were excluded because of excessive movement during scanning; therefore, 19 participants entered the final analysis.

For each participant, all functional volumes were realigned to the first volume acquired, corrected for slice-timing, normalized to the MNI (Montreal Neurological Institute) template implemented in SPM12, and smoothed with a 9 mm × 9 mm × 12 mm full-width at half-maximum Gaussian kernel.

The four conditions (SP, SNP, OP, and ONP) were modeled by convolving the respective stimulus timing vectors with the standard hemodynamic response function. Condition effects were estimated using a general linear model framework, and region-specific effects were investigated with linear contrasts comparing the four experimental conditions. For each volunteer, the Pain/NoPain labeling of the stimuli for the model regressors followed the answer that the subject provided. Group random-effects analyses were performed by entering the individual contrast images corresponding to the effects of interest into separate one-sample *t*-tests. A double statistical threshold (single-voxel statistics and spatial extent) was used to achieve a combined experiment-wise (i.e., corrected for multiple comparisons) significance level of α < 0.05, as computed by 3dClustSim AFNI routine^4^, with the “-acf” option.

Additional parametric analyses were performed to map regions whose activity was related to: (a) the actual intensity of perceived pain following P stimuli, according to the subjective ratings provided by the volunteers during the video clip recording session (obviously limited to the SP condition) and (b) the putative pain estimated by observing the facial expression during a post-scanning evaluation (for all the SP and OP stimuli presented during the scanning session).

To evaluate the interaction between Pain and Self, we first conducted whole brain repeated measure ANOVAs, then we used the average beta values extracted from two different regions of interest (ROIs), i.e., 3 mm-radius spheres centered in aMCC (*x* = 6; *y* = 20; *z* = 26) and right AI (*x* = 39; *y* = 11; *z* = –2). These coordinates correspond to the peak value of clusters resulting from the SPvsOP contrast, and are very close to those identified in the literature when comparing the effects of actual noxious vs. non-noxious skin stimulation (e.g., Lui et al., 2008; Duerden and Albanese, 2013). The beta extraction was performed using MarsBaR (Brett et al., 2002).

Finally, regression analyses were implemented to identify brain regions showing a correlation with (i) the individual degree of interoceptive accuracy (heartbeat perception score), (ii) the assessed personality traits, (iii) the level of state and trait anxiety, and (iv) the tendency to catastrophize pain.

For all analyses, coordinates in Talairach space (Talairach and Tournoux, 1988) were obtained by applying the Matthew Brett correction (mni2tal^5^) to the SPM MNI coordinates.

## Results

### Behavioral Data and Questionnaires

Accuracy and response times were not significantly different between the four conditions considered (Friedman test); mean percentage and reaction times of correct responses were, respectively, 90.6% and 1,336 ms for SP; 85.6% and 1,339 ms for OP; 93% and 1,349 ms for SNP and 94.2% and 1,336 ms for ONP. The mean Empathy Quotient (EQ) was 46.63 with a SD of 10.00; the mean IRI score was 96.58 with a SD of 11.85, the mean TAS 20 score was 57.79 with a SD of 7.23, the mean PCS score was 30.00 with a SD of 2.28.

### fMRI Data

#### Interaction Between Self and Pain

**FIGURE 2:**
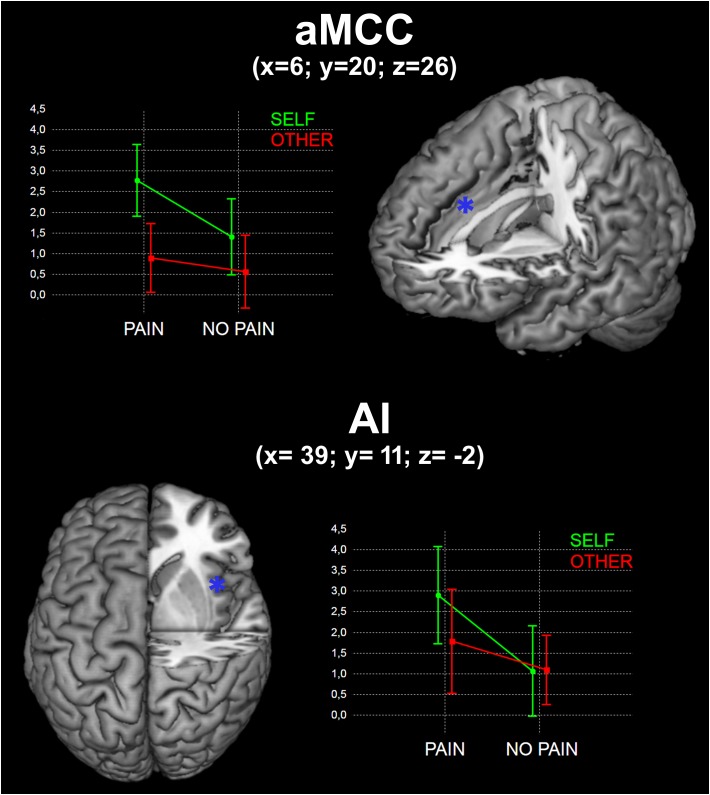
Results of the interaction between Self and Pain in the two identified ROIs (see Materials and Methods). x, y, and z are coordinates in the MNI space. Graphs represent mean beta values for the four experimental conditions.

No significant interaction was found in the whole-brain analysis. We observed a significant interaction between Self and Pain [(SPvsSNP) > (OPvsONP)] in the aMCC ROI [*F*_(1,__18)_ = 4.57; *p* < 0.05)], but not in the AI ROI [*F*_(1,__18)_ = 2.96; *p* > 0.05)] (**Figure 2**).

#### Pain vs. NoPain

**FIGURE 3:**
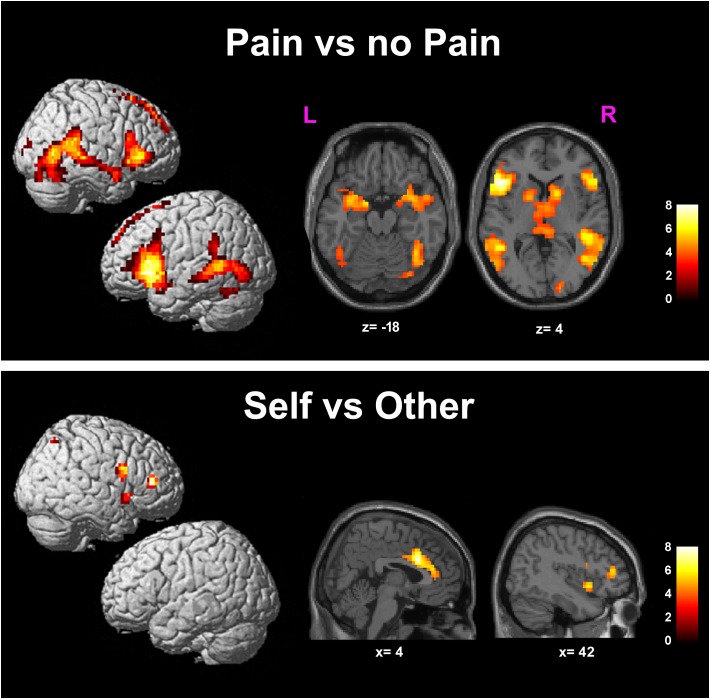
Results of the contrasts Pain vs. noPain (top) and Self vs. Other (bottom). Surface rendering (left) and regions of increased signal superimposed on slices of a standard structural T1 weighted brain (right). Color bars represent *T*-values. Statistical thresholds as in **Tables 1**, **2**, respectively. L, left; R, right; x and z are coordinates in the MNI space.

Greater BOLD responses for the observation of Pain compared to noPain (P vs. NP) stimuli were observed bilaterally in the inferior and middle occipital gyri, in the middle temporal gyrus (MTG), superior temporal gyrus (STG), supramarginal gyrus (SMG), amygdala, pre-supplementary motor area (preSMA), superior mesial frontal gyrus, anterior Middle Cingulate cortex (aMCC), inferior frontal gyrus (IFG), insular cortex, thalamus, putamen, and caudate nucleus. In the right hemisphere, the activation encompassed the fusiform gyrus (FG) (including the expected location of the fusiform face area, FFA (Kanwisher et al., 1997), and parts of the cerebellum (**Table 1** and **Figure 3**, top).

**Table 1:** Regions of increased signal for the contrast Pain vs. no Pain.

	Side	Cluster	Voxel level	MNI coordinates			Talairach coordinates		
Brain areas		*k*	*Z*	*x*	*y*	*z*	*x*	*y*	*z*
Bilateral inferior frontal gyrus (BA 47, 45, 44), anterior insula, amygdala, entorhinal cortex (BA 28, 34), l superior temporal gyrus (BA 38); r middle temporal gyrus (BA 21), superior temporal gyrus (BA 22), r fusiform gyrus (BA 37), inferior occipital gyrus, middle occipital gyrus (BA 19), thalamus, caudate nucleus, and putamen, cerebellum	r/l	2,943	5.25	−48	23	6	−48	23	4
			5.19	−39	20	−10	−39	19	−9
			5.06	51	−37	−2	50	−36	0
Middle and superior temporal gyri (BA 21, 22), supramarginal gyrus (BA 40), inferior, and middle occipital giri (BA 19),	l	392	5.11	−48	−76	−2	−48	−74	2
			4.76	−48	−49	6	−48	−47	8
			4.32	−54	−37	−2	−53	−36	0
Anterior-middle cingulate cortex (BA 24, 32), superior mesial frontal gyrus (BA 8, 9, 10), pre-supplementary motor area (BA 6)	r/l	597	5.01	6	17	62	6	19	56
			4.17	18	47	42	18	47	36
			4	3	47	38	3	47	33
Superior occipital gyrus (BA 17, 18)	l	32	3.9	18	−94	6	18	91	10
Fusiform gyrus (BA 37)	l	32	3.68	−45	−55	−22	−45	−54	−16
				−42	−67	−18	−42	−66	−12

*Cluster-size threshold k = 29 voxels, corrected at α < 0.05. r, right; l, left; BA, Brodmann area.*

#### Self vs. Other

Significantly greater responses for the observation of Self compared to Other stimuli (SvsO), were observed bilaterally in the ACC and aMCC, in the inferior and middle frontal gyri and in the AI on the right side. One additional cluster was found in the right parietal cortex (superior parietal lobule and angular gyrus) (**Table 2** and **Figure 3** bottom).

**Table 2:** Regions of increased signal for the contrast Self vs. Other.

	Side	Cluster	Voxel level	MNI coordinates			Talairach coordinates		
Brain areas		K	Z	x	y	z	x	y	z
Anterior (ACC) and anterior mid-cingulate (aMCC) cortex (BA 24, 32)	r/l	174	4.79	3	17	30	3	18	27
			4.01	0	35	18	0	35	15
Inferior frontal gyrus (BA 44)			3.95	0	26	22	0	26	19
Inferior and middle frontal gyri (BA 47, 10)	r	45	4.17	51	8	26	50	9	24
Anterior insula	r	57	3.97	39	41	14	39	40	11
Superior parietal lobule, angular gyrus (BA 7)	r	40	3.97	42	11	−6	42	10	−6
	l	40	3.83	24	−55	46	24	−51	45

*Cluster-size threshold k = 29 voxels, corrected at α < 0.05. r, right; l, left; BA, Brodmann area.*

#### Parametric and Regression Analyses

No significant cluster was found when using as regressors of interest in the analysis either the levels of perceived pain following SP stimuli during the video clip recording session, or the pain levels estimated by observing SP and OP stimuli.

No significant correlation was found between brain activity and (i) the assessed personality/behavioral scores (see above); (ii) the individual degree of interoceptive accuracy (heartbeat perception score).

## Discussion

To the best of our knowledge, this is the first study comparing the fMRI activations related to implicit processing of self and others’ facial expressions of pain.

### Interaction Between Self and Pain

We observed a significant interaction effect for the stimulus dimensions of Self and Pain on BOLD activity in the aMCC. It is well known from the literature that this region is involved in actual nociceptive processing and pain perception (Lui et al., 2008; Duerden and Albanese, 2013; Wager et al., 2013; Favilla et al., 2014). Although in this study we did not actually localize interoceptive or pain perception areas, nor did we explicitly manipulate any kind of embodied simulation, this finding suggests that, among the regions involved in pain and empathy for pain, aMCC has a crucial role in self/other discrimination and in self-focused affective reaction. It is worth mentioning that aMCC activity is increased during anticipation of pain (Porro et al., 2002), contributes to pain intensity coding (Lui et al., 2008; Favilla et al., 2014) and is positively correlated with the expectation of pain relief (Petrovic et al., 2005), further suggesting that aMCC is a key structure for the cognitive aspects of pain (Vogt, 2016).

The present findings add to the current body of knowledge the notion that, within these overlapping brain networks, the processing of self-experiences may still be differently modulated compared to others’ experiences.

### Pain Related Activations

We observed increased activation for the observation of Pain compared to noPain (PvsNP) face stimuli in pain related regions (Lui et al., 2008; Duerden and Albanese, 2013) such as aMCC, IFG, insular cortex and thalamus, as well as in a large bilateral array of cortical areas located in the occipital, temporal and frontal cortex.

These areas, with the exception of the aMCC, exhibited a response that was not significantly modulated by the Self/Other attribute of the stimulus, as they were below threshold in the statistical interaction map.

As described in the Introduction, previous fMRI studies consistently showed overlapping neural activations related to the direct experience of pain and to empathy for the pain of others (Lamm et al., 2011) in AI and aMCC (Carr et al., 2003; Singer et al., 2004, 2006; Saarela et al., 2007; Lamm et al., 2011; Zaki et al., 2016). These activations were found in studies presenting body parts (hands and feet) hit by painful stimuli, as well as faces expressing pain (Botvinick et al., 2005; Lamm et al., 2007; Saarela et al., 2007; Budell et al., 2010). Seeing hands or feet in painful situations (but not painful faces) also activated somatosensory cortex (Keysers et al., 2010) and facilitated motor programs associated with nociceptive pain (Avenanti et al., 2005). Brain responses to others’ pain in AI and aMCC correlate with subjective experiences of pain empathy (Saarela et al., 2007; Ochsner et al., 2008) and willingness to reduce others’ pain (Hein et al., 2010; Zaki and Ochsner, 2012). Moreover, brain responses to empathic pain diminish after placebo analgesia (Rütgen et al., 2015a,b).

The activation of an overlapping neural network for both the direct experience and the observation of others experiencing the same emotion has been interpreted within the framework of the “embodied simulation” perspective (Gallese, 2003; Gallese et al., 2004). Namely, the representations of one’s own emotions (as well as one’s own actions or sensations) driven by the direct experience, and the corresponding representations of observing others experiencing the same emotion, would share a common “bodily” format (Gallese and Sinigaglia, 2011). Data supporting this hypothesis come from studies demonstrating that AI and the ACC, key regions involved in the direct experience of disgust, were also implicated in the observation of others’ facial expressions of disgust (Wicker et al., 2003). It should be pointed out that there is not a clear demonstration that the same neural assemblies are active in the two conditions, but simply that they are spatially overlapping at the spatial resolution of conventional fMRI. The close connection between self and other experience in human brain is further demonstrated by neuropsychological findings showing that circumscribed lesions of the AI lead to selective deficits in experiencing and recognizing disgust (Calder et al., 2000).

As pointed out by Valentini (2010), it may be difficult in the present task to disentagle between pain recognition *per se* and empathic processing. The first process may be necessary but not sufficient to elicit empathy.

### Self-Related Activations

Observing one’s own face vs. another’s face (i.e., the SvsO contrast) evoked activations in bilateral ACC and aMCC, and in right AI and IFG. These areas, with the exception of the aMCC, exhibited a response that was not significantly modulated by the Pain/Neutral attribute of the stimulus, as they were below threshold in the statistical interaction map. Regarding the salience hypothesis that has been advanced by Valentini and Koch (2012) to explain the higher responses related to self-processing, in our opinion this might explain at least in part the main effect (Self vs. Other), but it can hardly explain the interaction effect.

Ibáñez et al. (2011) found that early and late cortical ERP response between pain and no-pain were modulated only in the other-face priming condition. However, as mentioned in the Introduction, self-related affect can bias empathy. More specifically, the observation of another person in pain can evoke the aversive experience of personal distress, triggering a self-focused, aversive affective reaction associated with the desire to alleviate one’s own, but not the other’s, pain (Eisenberg, 2000). Recently, it has been demonstrated that overcoming biased empathic judgments is associated with increased activation in the right supramarginal gyrus, thus suggesting that the activity of this region is crucial for the self/other distinction (Silani et al., 2013; Steinbeis et al., 2015). The specific function of this area is still unknown, but this region is clearly segregated from the one engaged during self/other distinction in the motor or cognitive domain, which is located in the right temporo-parietal junction (Silani et al., 2013; Steinbeis et al., 2015). With respect to the distinction of processing self vs. others’ faces without specific emotions, previous neuroimaging results have shown that explicit self-face recognition activates a frontoparietal network mostly located on the right hemisphere (Uddin et al., 2005; Apps et al., 2012). However, a meta-analysis including both neuropsychological and neuroimaging results revealed that the network involved is widespread and includes the left fusiform gyrus, the bilateral middle, and IFG gyri and right precuneus (Platek et al., 2008).

The activations reported here in aMCC and in right AI, evoked by observing one’s own face vs. others’ faces in pain, are in line with those of previous studies showing stronger responses in the same regions associated with higher personal distress (Jackson et al., 2006), or in the aMCC following short-term training in empathy, leading to increased empathic responses and negative affect (Klimecki et al., 2014). Another study evaluated the effect of personal distress or empathic concern manipulating the perspective-taking instructions of the task (viewing facial expressions of pain with the instruction to imagine the feelings of the other or to imagine oneself to be in the other’s situation), showing stronger responses related to personal distress in middle insula (Lamm et al., 2007).

Recently, Tania Singer and her group compared the neural activations for the response of “feeling as” (empathic resonance) and “feeling for” (compassion) by means of an event related fMRI study (Klimecki et al., 2014). Short-term training in empathic resonance increased negative affect and was associated with activations in a network comprising AI, aMCC, temporal gyrus, dorsolateral prefrontal cortex, and parts of basal ganglia. Compassion training decreased negative affect back to baseline levels and increased positive affect: it was associated with increased brain activations in a non-overlapping brain network comprising medial orbitofrontal cortex, pregenual ACC and striatum, a network previously associated with positive affect (Kringelbach and Berridge, 2009), reward and affiliation (Strathearn et al., 2009; Haber and Knutson, 2010).

### Regression Analyses

In the present study we found no significant correlation between brain activity and the individual degree of interoceptive awareness, the assessed personality traits, measures of individual empathy, level of anxiety and the tendency to catastrophize pain. Few neuroimaging studies evaluated similar correlations effects and have produced inconclusive results. In particular, correlations with trait and state measures of empathic concern and personal distress have generally been extremely weak: a few early studies reported a correlation between empathic concern and AI activation (Singer et al., 2004, 2006; Lamm et al., 2007); however, others have failed to replicate these results (see Lamm et al., 2011 for a review). It is clearly possible that negative results are due to the fact that these personality measures do not have a simple linear correlation with the BOLD brain activity involved in empathy for pain. Alternatively, the lack of a significant effect may be due to the noisy nature of the psychometric measures, to a large interindividual variability in the ability for introspectively accessing and reporting emotional states, and to complex, non-linear relationships between phenomenological and fMRI data.

### Methodological Considerations

In the present study we aimed at evaluating the impact of the self-other distinction on empathy related processes. In order to avoid the use of a passive paradigm, which is suboptimal because of potential drops in attentional engagement and task attendance by the subjects, two possible options were to ask participants to evaluate either the presence of pain, or the identity of the people represented in the pictures.

We opted for an explicit, rather than an implicit evaluation of pain for two reasons. First, the majority of fMRI studies on empathy used explicit tasks. In social cognition implicit and explicit processing are thought to be intrinsically different with distinct functions, sometimes complementary and sometimes oppositional (see Frith and Frith, 2008, for a review). Thus, asking participants to implicitly process pain would have not allowed us to compare our study with previous ones. The second issue regards the complexity of the task. It has been shown that recognizing oneself is more accurate (and easier) than recognizing others (see for example Apps et al., 2012). The explicit evaluation of pain, on the other hand, does not require different resources for processing self or others’ faces, as demonstrated by our behavioral results.

Possible limitations of our study are due to the fact that we compared self and others’ faces: for each participant, self face was repeated more often than any other single face, beside the fact that one’s own face is more familiar than any other face. However, in everyday life it is not common to see oneself expressing pain; it is rather more likely to see other people in pain. These issues make our stimuli inherently difficult to balance. Further studies performed manipulating the frequency of presentation of self faces might address these issues.

Finally, as noted in Methods, we decided to use only female volunteers, given the well-known differences between genders both in expressivity (LaFrance and Banaji, 1992; Hall et al., 2000), and in empathic behavior (Klein and Hodges, 2001; Singer et al., 2006): further studies are required to verify whether the present results are gender-specific.

## Conclusion

Within the largely overlapping neural network of pain-related regions for the implicit processing of self and others’ facial expressions of pain, self-related stimuli induced generally higher activations despite their lower ecological plausibility. This effect could not be ascribed to overt processing of self-related stimuli since we used an implicit task, requiring subjects to attend to the affective content of the stimuli, rather than to the identity of the portrayed person. Self-related effects were greater for pain-related stimuli in aMCC, a medial-wall region critical for pain perception (Duerden and Albanese, 2013; Favilla et al., 2014) and empathic resonance (Klimecki et al., 2014), supporting the hypothesis that empathic recognition is likely to be related to implicit simulation of painful experiences.

## Author Contributions

FB, FL, MAr, and MAm designed the study and carried out the experiments. FB, MAr, MAm, DB, and SR analyzed the data. FB, FL, MAr, MAm, GP, and CAP wrote the paper, which was approved by all authors. FL, VG, and CAP supervised the project.

## Conflict of Interest Statement

The authors declare that the research was conducted in the absence of any commercial or financial relationships that could be construed as a potential conflict of interest. The reviewer SCS and handling Editor declared their shared affiliation at time of review.

## Footnotes

1 http://virtualdubmod.sourceforge.net/

2 https://www.curdes.com/

3 https://digilander.libero.it/marco_serafini/stimoli_video/

4 https://afni.nimh.nih.gov/pub/dist/doc/program_help/3dClustSim.html

5 http://www.mrc-cbu.cam.ac.uk/Imaging/mnispace.html

## References

[B1] Albiero P. Ingoglia S. Lo Coco A. (2006). Contributo all’adattamento italiano dell’interpersonal reactivity index. *Test. Psicom. Metodol.* 13 107–125.

[B2] Ambrosecchia M. Ardizzi M. Russo E. Ditaranto F. Speciale M. Vinai P. (2017). Interoception and autonomic correlates during social interactions. Implications for anorexia. *Front. Hum. Neurosci.* 11:219. 10.3389/fnhum.2017.00219 28567008 PMC5434670

[B3] Apps M. A. J. Tajadura-Jiménez A. Turley G. Tsakiris M. (2012). The different faces of one’s self: an fMRI study into the recognition of current and past self-facial appearances. *Neuroimage* 63 1720–1729. 10.1016/j.neuroimage.2012.08.053 22940117 PMC3772343

[B4] Ardizzi M. Ambrosecchia M. Buratta L. Ferri F. Peciccia M. Donnari S. (2016). Interoception and positive symptoms in schizophrenia. *Front. Hum. Neurosci.* 10:379. 10.3389/fnhum.2016.00379 27512369 PMC4961721

[B5] Avenanti A. Bueti D. Galati G. Aglioti S. M. (2005). Transcranial magnetic stimulation highlights the sensorimotor side of empathy for pain. *Nat. Neurosci.* 8 955–960. 10.1038/nn1481 15937484

[B6] Batson C. D. (2011). “These things called empathy,” in *TheSocial Neuroscience of Empathy*, eds Decety J. Ickes W. (Cambridge, MA: The MIT Press), 3–1.

[B7] Batson C. D. Fultz J. Schoenrade P. A. (1987). Distress and empathy: two qualitatively distinct vicarious emotions with different motivational consequences. *J. Pers.* 55 19–39. 10.1111/j.1467-6494.1987.tb00426.x 3572705

[B8] Botvinick M. Jha A. P. Bylsma L. M. Fabian S. A. Solomon P. E. Prkachin K. M. (2005). Viewing facial expressions of pain engages cortical areas involved in the direct experience of pain. *Neuroimage* 25 312–319. 10.1016/j.neuroimage.2004.11.043 15734365

[B9] Brett M. Anton J. Valabregue R. Poline J. (2002). “Region of interest analysis using an SPM toolbox,” in *Proceedings of the 8th International Conference on Functional Mapping of the Human Brain, Available on CD-ROM in NeuroImage* Vol. 16 Sendai.

[B10] Budell L. Jackson P. Rainville P. (2010). Neuroimage Brain responses to facial expressions of pain: emotional or motor mirroring? *Neuroimage* 53 355–363. 10.1016/j.neuroimage.2010.05.037 20510372

[B11] Calder A. J. Keane J. Manes F. Antoun N. Young A. W. (2000). Impaired recognition and experience of disgust following brain injury. *Nat. Neurosci.* 3 1077–1078. 10.1038/80586 11036262

[B12] Caretti V. La Barbera D. Craparo G. (2005). “La Toronto Alexithymia Scale (TAS-20),” in *Alessitimia, Valutazione e Trattamento*, eds Caretti V. Barbera D. La (Roma: Astrolabio Ubaldini), 17–23.

[B13] Carr L. Iacoboni M. Dubeau M. C. Mazziotta J. C. Lenzi G. L. Ochsner K. N. (2003). Neural mechanisms of empathy in humans: a relay from neural systems for imitation to limbic areas. *Proc. Natl. Acad. Sci. U.S.A.* 100 5497–5502. 10.1007/s00429-010-0251-253 12682281 PMC154373

[B14] Carver C. S. White T. L. (1994). Behavioral inhibition, behavioral activation, and affective responses to impending reward and punishment: the BIS/BAS scales. *J. Pers. Soc. Psychol.* 67 319–333. 10.1037/0022-3514.67.2.319

[B15] Corradi-Dell’Acqua C. Hofstetter C. Vuilleumier P. (2011). Felt and seen pain evoke the same local patterns of cortical activity in insular and cingulate cortex. *J. Neurosci.* 31 17996–18006. 10.1523/JNEUROSCI.2686-11.2011 22159113 PMC6634156

[B16] Corradi-Dell’Acqua C. Tusche A. Vuilleumier P. Singer T. (2016). Cross-modal representations of first-hand and vicarious pain, disgust and fairness in insular and cingulate cortex. *Nat. Commun.* 7:10904. 10.1038/ncomms10904 26988654 PMC4802033

[B17] Craig K. D. Prkachin K. M. Grunau R. V. E. (2001). “The facial expression of pain,” in *Handbook of Pain Assessment*, eds Turnk R. Melzack D. C. (New York, NY: Guilford Press).

[B18] Davis M. H. (1983). A mulitdimensional approach to individual differences in empathy. *J. Pers. Soc. Psychol.* 44 113–126. 10.1037/0022-3514.44.1.113

[B19] Duerden E. G. Albanese M. C. (2013). Localization of pain-related brain activation: a meta-analysis of neuroimaging data. *Hum. Brain Mapp.* 34 109–149. 10.1002/hbm.21416 22131304 PMC6869965

[B20] Dunn B. D. Galton H. C. Morgan R. Evans D. Oliver C. Meyer M. (2010). Listening to your heart. How interoception shapes emotion experience and intuitive decision making. *Psychol. Sci.* 21 1835–1844. 10.1177/0956797610389191 21106893

[B21] Eisenberg N. (2000). Emotion, regulation, and moral development. *Annu. Rev. Neurosci.* 51 665–697. 10.1146/annurev.psych.51.1.665 10751984

[B22] Fan Y. Duncan N. W. de Greck M. Northoff G. (2011). Neuroscience and biobehavioral reviews is there a core neural network in empathy? An fMRI based quantitative. *Neurosci. Biobehav. Rev.* 35 903–911. 10.1016/j.neubiorev.2010.10.009 20974173

[B23] Favilla S. Huber A. Pagnoni G. Lui F. Facchin P. Cocchi M. (2014). Ranking brain areas encoding the perceived level of pain from fMRI data. *Neuroimage* 90 153–162. 10.1016/j.neuroimage.2014.01.001 24418504

[B24] Frith C. D. Frith U. (2008). Implicit and explicit processes in social cognition. *Neuron* 60 503–510. 10.1016/j.neuron.2008.10.032 18995826

[B25] Gallese V. (2003). The manifold nature of interpersonal relations: the quest for a common mechanism. *Philos. Trans. R. Soc. London. Ser. B Biol. Sci.* 358 517–528. 12689377 10.1098/rstb.2002.1234 PMC1693141

[B26] Gallese V. Keysers C. Rizzolatti G. (2004). A unifying view of the basis of social cognition. *Trends Cogn. Sci.* 8 396–403. 10.1016/j.tics.2004.07.002 15350240

[B27] Gallese V. Sinigaglia C. (2011). What is so special about embodied simulation? *Trends Cogn. Sci.* 15 512–519. 10.1016/j.tics.2011.09.003 21983148

[B28] Haber S. N. Knutson B. (2010). The reward circuit: linking primate anatomy and human imaging. *Neuropsychopharmacology* 35 4–26. 10.1038/npp.2009.129 19812543 PMC3055449

[B29] Hall J. Carter J. Horgan T. (2000). “Gender differences in nonverbal communication of emotion,” in *Gender and Emotion: Social Psychological Perspectives (Studies in Emotion and Social Interaction*, ed. Fischer A. (Cambridge: Cambridge University Press), 97–117. 10.1017/CBO9780511628191.006

[B30] Hein G. Silani G. Preuschoff K. Batson C. D. Singer T. (2010). Article neural responses to ingroup and outgroup members ’ suffering predict individual differences in costly helping. *Neuron* 68 149–160. 10.1016/j.neuron.2010.09.003 20920798

[B31] Henrich J. Hammerstein P. Press T. F. Sigmund K. Boyd R. Szathmary E. (2007). Embodying emotion. *Science* 316 1002–1005. 10.1126/science.1136930 17510358

[B32] Ibáñez A. Hurtado E. Lobos A. Escobar J. Trujillo N. Baez S. (2011). Subliminal presentation of other faces (but not own face) primes behavioral and evoked cortical processing of empathy for pain. *J. Brain Res.* 1398 72–85. 10.1016/j.brainres.2011.05.014 21624566

[B33] Jackson P. L. Brunet E. Meltzoff A. N. Decety J. (2006). Empathy examined through the neural mechanisms involved in imagining how I feel versus how you feel pain. *Neuropsychologia* 44 752–761. 10.1016/j.neuropsychologia.2005.07.015 16140345

[B34] Kanwisher N. McDermott J. Chun M. M. (1997). The fusiform face area: a module in human extrastriate cortex specialized for face perception. *J. Neurosci.* 17 4302–4311. 10.1523/JNEUROSCI.17-11-04302.1997 9151747 PMC6573547

[B35] Keysers C. Kaas J. H. Gazzola V. (2010). Somatosensation in social perception. *Nat. Rev. Neurosci.* 11 417–428. 10.1038/nrn2919 20445542

[B36] Klein K. J. K. Hodges S. D. (2001). Gender differences, motivation, and empathic accuracy: when it pays to understand. *Pers. Soc. Psychol. Bull.* 27 720–730. 10.1177/0146167201276007

[B37] Klimecki O. M. Leiberg S. Ricard M. Singer T. (2014). Differential pattern of functional brain plasticity after compassion and empathy training. *Soc. Cogn. Affect. Neurosci.* 9 873–879. 10.1093/scan/nst060 23576808 PMC4040103

[B38] Kringelbach M. L. Berridge K. C. (2009). Towards a functional neuroanatomy of pleasure and happiness. *Trends Cogn. Sci.* 13 479–487. 10.1016/j.tics.2009.08.006 19782634 PMC2767390

[B39] LaFrance M. Banaji M. (1992). “Toward a reconsideration of the gender-emotion relationship,” in *Review of Personality and Social Psychology: Emotions and Social Behavior* Vol. 14 ed. Clark M. S. (Newbury Park, CA: Sage), 178–202.

[B40] Lamm C. Batson C. D. Decety J. (2007). The neural substrate of human empathy: effects of perspective-taking and cognitive appraisal. *J. Cogn. Neurosci.* 19 42–58. 10.1162/jocn.2007.19.1.42 17214562

[B41] Lamm C. Decety J. Singer T. (2011). Meta-analytic evidence for common and distinct neural networks associated with directly experienced pain and empathy for pain. *Neuroimage* 54 2492–2502. 10.1016/j.neuroimage.2010.10.014 20946964

[B42] Lui F. Duzzi D. Corradini M. Serafini M. Baraldi P. Porro C. A. (2008). Touch or pain? Spatio-temporal patterns of cortical fMRI activity following brief mechanical stimuli. *Pain* 138 362–374. 10.1016/j.pain.2008.01.010 18313223

[B43] Monticone M. Baiardi P. Ferrari S. Foti C. Mugnai R. Pillastrini P. (2012). Development of the Italian version of the pain catastrophising scale (PCS-I): cross-cultural adaptation, factor analysis, reliability, validity and sensitivity to change. *Qual. Life Res.* 21 1045–1050. 10.1007/s11136-011-0007-4 21912846

[B44] Ochsner K. N. Zaki J. Hanelin J. Ludlow D. H. Knierim K. Ramachandran T. (2008). Neural mechanisms of empathy in humans: a relay from neural systems for imitation to limbic areas. *Proc. Natl. Acad. Sci. U.S.A.* 3 5497–5502. 10.1007/s00429-010-0251-253 12682281 PMC154373

[B45] Oldfield R. C. (1971). The assessment and analysis of handedness: the Edinburgh inventory. *Neuropsychologia* 9 97–113. 10.1016/0028-3932(71)90067-4 5146491

[B46] Petrovic P. Dietrich T. Fransson P. Andersson J. Carlsson K. Ingvar M. (2005). Placebo in emotional processing - Induced expectations of anxiety relief activate a generalized modulatory network. *Neuron* 46 957–969. 10.1016/j.neuron.2005.05.023 15953423

[B47] Platek S. M. Wathne K. Tierney N. G. Thomson J. W. (2008). Neural correlates of self-face recognition: an effect-location meta-analysis. *Brain Res.* 1232 173–184. 10.1016/j.brainres.2008.07.010 18656465

[B48] Pollatos O. Herbert B. M. Matthias E. Schandry R. (2007). Heart rate response after emotional picture presentation is modulated by interoceptive awareness. *Int. J. Psychophysiol.* 63 117–124. 10.1016/j.ijpsycho.2006.09.003 17137662

[B49] Pollatos O. Kurz A.-L. Albrecht J. Schreder T. Kleemann A. M. Schöpf V. (2008). Reduced perception of bodily signals in anorexia nervosa. *Eat. Behav.* 9 381–388. 10.1016/j.eatbeh.2008.02.001 18928900

[B50] Porro C. A. Baraldi P. Pagnoni G. Serafini M. Facchin P. Maieron M. (2002). Does anticipation of pain affect cortical nociceptive systems? *J. Neurosci.* 22 3206–3214. 10.1523/JNEUROSCI.22-08-03206.2002 11943821 PMC6757517

[B51] Preti A. Vellante M. Baron-Cohen S. Zucca G. Petretto D. R. Masala C. (2011). The empathy quotient: a cross-cultural comparison of the Italian version. *Cogn. Neuropsychiatry* 16 50–70. 10.1080/13546801003790982 20737328

[B52] Rütgen M. Seidel E.-M. Rieèanskı I. Lamm C. (2015a). Reduction of empathy for pain by placebo analgesia suggests functional equivalence of empathy and first-hand emotion experience. *J. Neurosci.* 35 8938–8947. 10.1523/JNEUROSCI.3936-14.2015 26063925 PMC6605205

[B53] Rütgen M. Seidel E.-M. Silani G. Rieèanskı I. Hummer A. Windischberger C. (2015b). Placebo analgesia and its opioidergic regulation suggest that empathy for pain is grounded in self pain. *Proc. Natl. Acad. Sci. U.S.A.* 112 E5638–E5646. 10.1073/pnas.1511269112 26417092 PMC4611649

[B54] Saarela M. V. Hlushchuk Y. Williams A. C. D. C. Schürmann M. Kalso E. Hari R. (2007). The compassionate brain: humans detect intensity of pain from another’s face. *Cereb. Cortex* 17 230–237. 10.1093/cercor/bhj141 16495434

[B55] Sanavio E. Bertolotti G. Michielin P. Vidotto G. Zotti A. (1997). *CBA 2.0: Cognitive Behavioural Assessment: Scale Primarie.* Florence: Organizzazioni Speciali. 10.1016/0005-7916(90)90045-m 2197296

[B56] Schandry R. (1981). Heart beat perception and emotional experience. *Psychophysiology* 18 483–488. 10.1111/j.1469-8986.1981.tb02486.x 7267933

[B57] Silani G. Lamm C. Ruff C. C. Singer T. (2013). Right supramarginal gyrus is crucial to overcome emotional egocentricity bias in social judgments. *J. Neurosci.* 33 15466–15476. 10.1523/JNEUROSCI.1488-13.2013 24068815 PMC6618458

[B58] Singer T. Seymour B. O’Doherty J. Kaube H. Dolan R. J. Frith C. D. (2004). Empathy for pain involves the affective but not sensory components of pain. *Science* 303 1157–1162. 10.1126/science.1093535 14976305

[B59] Singer T. Seymour B. O’Doherty J. P. Stephan K. E. Dolan R. J. Frith C. D. (2006). Empathic neural responses are modulated by the perceived fairness of others. *Nature* 439 466–469. 10.1038/nature04271 16421576 PMC2636868

[B60] Steinbeis N. Bernhardt B. C. Singer T. (2015). Age-related differences in function and structure of rSMG and reduced functional connectivity with DLPFC explains heightened emotional egocentricity bias in childhood. *Soc. Cogn. Affect. Neurosci.* 10 302–310. 10.1093/scan/nsu057 24771281 PMC4321629

[B61] Strathearn L. Fonagy P. Amico J. Montague P. R. (2009). Adult attachment predicts maternal brain and oxytocin response to infant Cues. *Neuropsychopharmacology* 34 2655–2666. 10.1038/npp.2009.103 19710635 PMC3041266

[B62] Talairach J. Tournoux P. (1988). *Co-Planar Stereotaxic Atlas of the Human Brain.* New York, NY: Thieme Medical Publisher, Inc.

[B63] Uddin L. Q. Kaplan J. T. Molnar-Szakacs I. Zaidel E. Iacoboni M. (2005). Self-face recognition activates a frontoparietal “mirror” network in the right hemisphere: an event-related fMRI study. *Neuroimage* 25 926–935. 10.1016/j.neuroimage.2004.12.018 15808992

[B64] Valentini E. (2010). The role of anterior insula and anterior cingulate in empathy for pain. *J. Neurophysiol.* 104 584–586. 10.1152/jn.00487.2010 20554847

[B65] Valentini E. Koch K. (2012). Fine-grained analysis of shared neural circuits between perceived and observed pain: implications for the study of empathy for pain. *J. Neurophysiol.* 108 1805–1807. 10.1152/jn.00181.2012 22623489

[B66] Vogt B. A. (2016). Midcingulate cortex: structure, connections, homologies, functions and diseases. *J. Chem. Neuroanat.* 74 28–46. 10.1016/j.jchemneu.2016.01.010 26993424

[B67] Wager T. D. Atlas L. Y. Lindquist M. A. Roy M. Woo C.-W. Kross E. (2013). An fMRI-based neurologic signature of physical pain. *N. Engl. J. Med.* 368 1388–1397. 10.1056/NEJMoa1204471 23574118 PMC3691100

[B68] Wicker B. Keysers C. Plailly J. Royet J. P. Gallese V. Rizzolatti G. (2003). Both of us disgusted in My insula: the common neural basis of seeing and feeling disgust. *Neuron* 40 655–664. 10.1016/S0896-6273(03)00679-2 14642287

[B69] Zaki J. Ochsner K. N. (2012). The neuroscience of empathy: progress, pitfalls and promise. *Nat. Neurosci.* 15 1–6. 10.1038/nn.3085 22504346

[B70] Zaki J. Wager T. D. Singer T. Keysers C. Gazzola V. (2016). The anatomy of suffering: understanding the relationship between nociceptive and empathic pain. *Trends Cogn. Sci.* 20 249–259. 10.1016/j.tics.2016.02.003 26944221 PMC5521249

